# Effect of material deprivation on Epstein–Barr virus infection in Hodgkin's disease in the West Midlands

**DOI:** 10.1038/sj.bjc.6690398

**Published:** 1999-05-01

**Authors:** K Flavell, C Constandinou, D Lowe, K Scott, C Newey, D Evans, A Dutton, S Simmons, R Smith, J Crocker, L S Young, P Murray

**Affiliations:** School of Health Sciences, University of Wolverhampton, Wolverhampton WV1 1DJ, UK; Department of Histopathology, New Cross Hospital, Wolverhampton, WV10 0QP, UK; Department of Histopathology, Russells Hall Hospital, Dudley, West Midlands DY1 2HQ, UK; West Midlands Cancer Intelligence Unit, The Public Health Building, The University of Birmingham, Birmingham B15 2TT, UK; Department of Histopathology, Birmingham Heartland's Hospital, Bordesley Green East, Birmingham B9 5SS, UK; Institute for Cancer Studies, University of Birmingham, Edgbaston, Birmingham B15 2TT, UK

**Keywords:** Epstein–Barr virus, Hodgkin's disease, Townsend score, material deprivation

## Abstract

We have used Townsend scores from postcode data to compare levels of material deprivation and Epstein–Barr virus (EBV)-positivity for 223 patients diagnosed with Hodgkin's disease (HD) in the period 1981–1997. The presence of EBV in HD tumours was determined using in situ hybridization to target the abundantly expressed EBV early RNAs. EBV was detected in the malignant Hodgkin and Reed–Sternberg cells in 47/223 HD cases (21%). There was found to be a tendency for higher Townsend scores (indicative of higher levels of material deprivation) in EBV-positive HD patients, but this association was not statistically significant. When various subgroups of patients from the study were examined separately the indication of higher Townsend scores in EBV-positive patients was found to be more marked for patients with mixed cellularity disease (*P*= 0.09) and for females (*P*= 0.03). The results of this study suggest that differences in the level of material deprivation are important in determining the likelihood of EBV-positive HD in the UK, particularly for certain subgroups of patients. It is not known what specific socioeconomic factors are responsible for these differences, although alterations in the timing or rate of primary EBV infection, or decline in the level of EBV-specific immunity, may be important. © 1999 Cancer Research Campaign


*British Journal of Cancer* (1999) **80**(3/4), 604–608
© 1999 Cancer Research Campaign
Article no. bjoc.1998.0398

### Effect of material deprivation on Epstein–Barr virus infection in Hodgkin's disease in the West Midlands

**K Flavell[1], C Constandinou[1], D Lowe[1], K Scott[2], C Newey[3], D Evans[3], A Dutton[1], S Simmons[1], R Smith[4], J Crocker[5], LS Young[6] and P Murray[1,6]**

[1]School of Health Sciences, University of Wolverhampton, Wolverhampton WV1 1DJ, UK; [2]Department of Histopathology, New Cross Hospital, Wolverhampton, WV10 0QP, UK; [3]Department of Histopathology, Russells Hall Hospital, Dudley, West Midlands DY1 2HQ UK; [4]West Midlands Cancer Intelligence Unit, The Public Health Building, The University of Birmingham, Birmingham B15 2TT, UK; [5]Department of Histopathology, Birmingham Heartland's Hospital, Bordesley Green East, Birmingham B9 5SS, UK; [6]Institute for Cancer Studies, University of Birmingham, Edgbaston, Birmingham B15 2TT, UK

**Summary** We have used Townsend scores from postcode data to compare levels of material deprivation and Epstein–Barr virus (EBV)-positivity for 223 patients diagnosed with Hodgkin's disease (HD) in the period 1981–1997. The presence of EBV in HD tumours was determined using in situ hybridization to target the abundantly expressed EBV early RNAs. EBV was detected in the malignant Hodgkin and Reed–Sternberg cells in 47/223 HD cases (21%). There was found to be a tendency for higher Townsend scores (indicative of higher levels of material deprivation) in EBV-positive HD patients, but this association was not statistically significant. When various subgroups of patients from the study were examined separately the indication of higher Townsend scores in EBV-positive patients was found to be more marked for patients with mixed cellularity disease ($P = 0.09$) and for females ($P = 0.03$). The results of this study suggest that differences in the level of material deprivation are important in determining the likelihood of EBV-positive HD in the UK, particularly for certain subgroups of patients. It is not known what specific socioeconomic factors are responsible for these differences, although alterations in the timing or rate of primary EBV infection, or decline in the level of EBV-specific immunity, may be important.

**Keywords:** Epstein–Barr virus; Hodgkin's disease; Townsend score; material deprivation

The Epstein–Barr virus (EBV), a B-lymphotropic human herpesvirus that infects the majority of the world's adult population, has been linked to a number of human malignancies, including Burkitt's lymphoma, nasopharyngeal carcinoma and, more recently, Hodgkin's disease (HD). The observation that persons with a history of infectious mononucleosis have a two- to threefold increased risk of HD (Gutensohn and Cole, 1980) and the detection of elevated levels of antibodies to viral antigens in HD patients prior to, or at the time of, diagnosis (Mueller et al, 1989; Levine et al, 1994; Alexander et al, 1995) provided the first evidence to suggest a causal role for EBV in HD.

Direct evidence for the association was provided by the localization of EBV DNA to the malignant Hodgkin and Reed–Sternberg (HRS) cells of HD (Weiss et al, 1987). Subsequently, the presence of latent EBV infection of tumour cells in HD was confirmed by in situ hybridization and immunohistochemical assays that targeted the highly abundant Epstein–Barr virus early RNAs (EBERs) and the latent membrane protein-1 (LMP1) (Anagnostopoulos et al, 1989; Wu et al, 1990; Pallesen et al, 1991; Murray et al, 1992). However, the frequency of EBV-positivity in HD tumours was found to be dependent upon several factors including age, sex and country of residence. EBV is more commonly associated with the mixed cellularity subtype and less frequently with the other forms of this disease (Pallesen et al, 1991; Murray et al, 1992; Jarrett et al, 1996; Glaser et al, 1997). In addition, Hodgkin's disease at older ages (> 55 years) and in children, especially those under 10 years, is more likely to be EBV-associated than is HD in young adults (Jarrett et al, 1991, 1996; Glaser et al, 1997; Armstrong et al, 1998*a*). The infrequent association of EBV with HD in young adults has prompted the suggestion that a second virus may be involved, although there is little evidence to support this at present (Armstrong et al, 1998*b*).

EBV-positive HD tumours also appear to be less common in developed populations, with percentages of between 30 and 50% for North American and European cases (Wu et al, 1990; Weiss et al, 1991; Herbst et al, 1992; Hummel et al, 1992), 57% for HD in China (Zhou et al, 1993), but with much higher rates (up to 100%) in underdeveloped countries such as Peru (Chang et al, 1993) and Kenya (Leoncini et al, 1996; Weinreb et al, 1996*a*, 1996*b*). In the present study, we have investigated whether in the UK, the frequency of EBV infection in Hodgkin's tumours is higher in underprivileged populations.

### MATERIALS AND METHODS

### Patients

Paraffin wax-embedded tissue blocks from patients with histologically confirmed Hodgkin's disease diagnosed between 1981 and 1997 were retrieved from the files of a number of hospitals in the West Midlands Region, including Russells Hall Hospital, Dudley; Good Hope Hospital, Sutton Coldfield; New Cross Hospital, Wolverhampton; and the Manor Hospital, Walsall. There were no specific exclusion criteria and children as well as adults were

*Received 17 July 1998*
*Revised 16 October 1998*
*Accepted 21 October 1998*

*Correspondence to*: PG Murray included. When patients from the present study were grouped into age ranges (0–14, 15–34, 35–54, > 55 years) and incidence rates compared to those in the West Midlands region there was found to be no significant difference, thus indicating that our patient group was representative of the West Midlands as a whole. Date of diagnosis and date of birth, sex and postcode data were collected anonymously for each patient.

### Histological review

Haematoxylin and eosin-stained slides from all tissue blocks were prepared and reclassified according to the REAL classification system (Harris et al, 1994) by a single pathologist (JC). Four-micrometer paraffin-sections were prepared, deparaffinized and washed in TBS pH 7.6.

### Detection of latent EBV infection

In situ hybridization for the detection of the EBERs was performed according to standard methodology (Barletta et al, 1993) and was used to detect the presence of latent EBV infection in all HD samples. Positive controls for EBER in situ hybridization included paraffin-wax sections of lymphoblastoid cell lines (LCLs) grown as solid tumours in SCID mice and a known EBER-positive HD case. U6 and sense control probes were included in all runs and their use has been previously described elsewhere (Barletta et al, 1993). Immunohistochemistry for LMP1 was performed on selected cases to confirm the presence of EBV infection in HRS cells. The standard APAAP method was employed and sections were microwave pre-treated as previously described (Murray et al, 1996*a*). Positive controls for LMP1 consisted of paraffin-wax sections of LCLs grown as solid tumours in SCID mice. Negative controls consisted of consecutive test sections in which primary antibody was replaced with non-immune serum of the same IgG subclass.

### Determination of material deprivation

Townsend scores matched to enumeration district and based on data from the 1991 OPCS census were generated from postcodes available for each patient. The Townsend score has been widely accepted as an indicator of material deprivation (Morris and Carstairs, 1991) and is calculated using statistical methods from the following four variables (Townsend et al, 1988; Bithell et al, 1995):

1. Percent of economically active residents (aged 16–64 for men, 16–59 for women) who are unemployed.
2. Percent of households without a car.
3. Percent of households not owner occupied.
4. Percent of households overcrowded (more than one person per room).

### Statistical methods

Tumour specimens were recorded as either EBV-positive (EBERs and LMP1 present within HRS cells) or EBV-negative (EBERs and LMP1 not detectable in HRS cells). EBV status was then compared with Townsend scores for all patients, and for several subgroups of patients based on gender, age and subtype. Mann–Whitney and $\chi^2$ tests and logistic regression methods were used to identify any significant differences between EBV-positive and negative groups in terms of Townsend score.

### RESULTS

### Detection of latent EBV infection in HD samples

The presence of latent EBV infection, as detected by in situ hybridization for the EBERs, was identified in HRS cells in 47/223 (21%) cases (EBV-positive group). The remaining 176 (79%) cases did not show the presence of the EBERs within HRS

**Table 1** Townsend scores for Hodgkin's disease patients, by subtype, age and gender

| | | EBV-positive | | | | EBV-negative | | | | Mann–Whitney test |
|---|---|---|---|---|---|---|---|---|---|---|
| | No. of patients | *n* (%) | 25th Centile | Median | 75th Centile | *n* | 25th Centile | Median | 75th Centile | *P*-Value |
| All patients | 223 | 47 (21) | –1.4 | 2.2 | 4.0 | 176 | –2.4 | 1.3 | 3.3 | 0.12 |
| All patients except LP and unknown subtypes | 172 | 39 (23) | –1.7 | 2.2 | 3.9 | 133 | –2.1 | 1.3 | 3.3 | 0.26 |
| All patients except LP & LD and unknown subtypes | 159 | 35 (22) | –0.5 | 2.2 | 4.0 | 124 | –2.4 | 1.3 | 3.1 | 0.06 |
| NS subtype only | 118 | 18 (15) | 0.6 | 2.4 | 4.0 | 100 | –2.0 | 1.5 | 3.3 | 0.22 |
| MC subtype only | 41 | 17 (41) | –1.5 | 2.2 | 4.0 | 24 | –3.9 | –0.5 | 2.8 | 0.09 |
| All male patients | 139 | 37 (27) | –1.9 | 2.0 | 3.9 | 102 | –1.8 | 1.1 | 3.7 | 0.62 |
| All female patients | 84 | 10 (12) | 1.8 | 3.2 | 5.2 | 74 | –3.1 | 1.3 | 3.1 | 0.03 |
| All patients 0–14 years | 7 | 0 (0) | – | – | – | 7 | – | –1.9 | – | – |
| All patients 15–34 years | 99 | 21 (21) | –1.3 | 2.2 | 4.0 | 78 | –2.8 | 1.2 | 3.1 | 0.18 |
| All patients 35–54 years | 40 | 9 (23) | – | 0.9 | – | 31 | –2.7 | 1.8 | 4.0 | 0.34 |
| All patients >55 years | 77 | 17 (22) | –0.5 | 2.7 | 4.8 | 60 | –1.2 | 1.1 | 3.8 | 0.14 |

LP: lymphocyte predominant, LD: lymphocyte depletion, NS: nodular sclerosis, MC: mixed cellularity.

© Cancer Research Campaign 1999

cells and will subsequently be referred to as the EBV-negative group. Immunohistochemistry for the LMP1 protein gave similar results to EBER in situ hybridization.

### Socioeconomic deprivation and EBV positivity

Townsend values ranged from –6.6 to +7.8. The majority of EBV-positive patients (33/47) had Townsend scores above zero (indicating higher levels of material deprivation), whereas relatively fewer (101/176) EBV-negative patients had scores above zero ($\chi^2$ test, $P$ = 0.11, i.e. not significant). Median Townsend scores for EBV-positive and EBV-negative groups were 2.2 and 1.3 respectively. The Mann–Whitney test gave a $P$-value of 0.12 (Table 1), also not significant.

Similar results were obtained when patients of unknown subtype together with those with lymphocyte predominant (LP) subtype were removed from the analysis ($P$ = 0.26) When the lymphocyte depletion (LD) subtype was also removed from the analysis, the Mann–Whitney test gave a $P$-value of 0.06 (i.e. of borderline significance).

The nodular sclerosis (NS) and mixed cellularity (MC) subtypes were also analysed separately. As expected, fewer NS cases were EBV-positive (18/118) when compared to MC patients (17/41). In both subtypes the trend towards higher Townsend scores for EBV-positive patients was maintained, particularly for MC patients ($P$ = 0.09). However, the differences were not of statistical significance in either subtype.

Differences in the level of material deprivation between EBV-positive and EBV-negative groups were also influenced by gender. Overall, male patients were more numerous in this study (139 male vs 84 female) and were more likely to be EBV-positive than female patients (37/139 = 27% male patients, and 10/84 = 12% female patients, were EBV-positive; $P < 0.05$). Although there was a tendency for higher Townsend scores in both male and female EBV-positive patients when compared to EBV-negative groups, this relationship was weaker for males ($P$ = 0.62), but achieved statistical significance for females ($P$ = 0.03). Further analysis of the female data revealed that the EBV-positive tumours were generally from older patients (median age 67 years), when compared to EBV-negative patients (median age 34 years) and were more likely to be of mixed cellularity subtype (50% of EBV-positive patients compared to only 10% for EBV-negative cases).

We were also interested to identify any differences in the groups in terms of age. EBV-positive and -negative patients were subdivided into age ranges: 0–14, 15–34, 35–54, > 55 years. Statistical analysis of the youngest age group was not valid due to low numbers. In none of the other age groups was there a statistically significant difference in Townsend scores between the EBV-positive and EBV-negative patients.

A stepwise logistic regression analysis was also performed with EBV status as the outcome or dependent variable. The predictor variables used were age (0–14, 15–34, 35–54, > 55 years), subtype (MC, NS, LP/LD), gender and Townsend scores. Patients with Hodgkin's disease of unknown subtype were excluded from this analysis. Subtype was the strongest predictor of EBV-positive status ($P$ = 0.0023). Gender then added to the prediction at the 5% level ($P$ = 0.03). At no stage did age-group appear to be an influential predictor, while Townsend values were of borderline significance as a predictor at the outset of the regression ($P$ = 0.15) and also after subtype group and gender had been allowed for ($P$ = 0.11).

When the logistic regression was re-run, excluding LP and LD subtypes, the main predictor of EBV-positive status was again subtype ($P < 0.001$), but with Townsend score in second place ($P$ = 0.03). After subtype had been allowed for, gender no longer appeared an influential predictor ($P$ = 0.18).

### DISCUSSION

Among persons under the age of 45, the age-incidence curve of Hodgkin's disease varies according to the level of socioeconomic development (MacMahon, 1966; Correa and O'Conor, 1971). In developed populations, the incidence of HD is low among children, increases sharply through adolescence, peaks at around 30 years, and then declines until the mid-40s (Gutensohn and Cole, 1981; Bernard et al, 1987). By contrast, in underdeveloped countries there is an early incidence peak in childhood that affects predominantly boys, and only a modest increase through adolescence. Taken together these patterns suggest that material deprivation in childhood is associated with a greater risk of paediatric HD, while HD in young adults is associated more with relative affluence. At older ages HD is the result of a decline in immune function, and this in turn may be related to deprivation. It has been suggested that HD may be a rare consequence of infection with a common virus, and that the probability of its occurrence increases when infection is delayed until adolescence or young adulthood (Shimkin, 1955).

Our results have demonstrated a tendency for higher Townsend scores in EBV-positive HD patients compared with EBV-negative cases, indicating that EBV-positive HD in the UK is more likely in patients from materially deprived areas. This relationship was strongest when the lymphocyte predominant and lymphocyte depletion subtypes were excluded from the analyses. Lymphocyte-predominant disease differs morphologically, immunophenotypically and clinically from classic HD (Linden et al, 1988; Harris et al, 1994; Stoler et al, 1995) and there is controversy over the morphological distinction between LD HD and anaplastic large cell lymphoma in which EBV is also implicated as a pathogen (Herbst et al, 1991; Stein et al, 1991; Frizzera, 1992; Hansmann and Kuppers, 1996).

The higher frequency of EBV-positive cases and the Townsend scores among the latter for the mixed cellularity subtypes of HD accords with results from underdeveloped populations in which MC HD has previously been reported to be proportionally more common and is frequently EBV-positive (Ambinder et al, 1993; Chang et al, 1993; Leoncini et al, 1996; Weinreb et al, 1996*a*, 1996*b*). Thus, our results suggest that, within this subtype, socioeconomic factors may be particularly important in determining EBV positivity.

When gender was examined, female patients with EBV-positive disease tended to have higher Townsend scores than the EBV-negative group ($P$ = 0.03). The pattern of occurrence was similar for male patients, but the difference was not statistically significant. This study also shows that males overall are more likely to develop EBV-positive HD than females ($P$ = 0.02), confirming previous studies from the UK (Murray et al, 1996*b*). Gender differences are also apparent when comparing developed and developing populations. Males, particularly boys with EBV-positive MC disease, tend to characterize HD in underdeveloped populations, whereas young adult females with EBV-negative NS disease are primarily responsible for the young adult peak observed in developed countries (Ambinder et al, 1993; Gulley et al, 1994; Jarrett et al, 1996; Glaser et al, 1997). It has been suggested that these differences may be due to variations in the

© Cancer Research Campaign 1999

childhood social environment, between boys and girls (Gutensohn and Cole, 1981). We can speculate that, for boys, risk factors for EBV-positive HD are often present, irrespective of the level of material deprivation, whereas for girls, they are only present under impoverished conditions. Thus, we might expect only minor effects of material deprivation on the frequency of EBV-positivity in males, but a much more dramatic effect in females.

When patients were grouped into the commonly accepted age ranges we were unable to find any significant differences in Townsend scores between EBV-positive and -negative groups although at ages 15–34 and > 55 (for which the numbers were largest) the pattern of occurrence for Townsend score by EBV status was similar to that for the total study group. Clearly, there is a need to examine further the possible impact of Townsend scores on EBV positivity within various age groups. Ethnic or racial differences have also been shown to affect the frequency of EBV positivity in HD. In one study, three of four Hispanic Americans, but only one of five blacks and four of ten Caucasians were EBV-positive (Ambinder et al, 1993). Analysis of a much larger series of HD cases by Gulley et al (1994) also showed an association with Hispanic ethnicity ($P < 0.001$). Several UK studies have shown that Asian children have a higher rate of HD, with an estimated relative risk of 2.09 (Stiller et al, 1991; Muir et al, 1992; Varghese et al, 1996), but data on the frequency of EBV positivity in these tumours is not available. We were not able to determine ethnic or racial origin in our group of patients and the possibility that these factors may confound any association between EBV positivity in HD and material deprivation cannot be ruled out. Clearly, further analysis taking into account ethnic or racial differences is required. We have used computer-derived Townsend scores, matched for enumeration districts identified from post-codes as a measure of material deprivation for each patient. Studies of 'small areas' have been recognized as an acceptable basis for investigating the influence of material deprivation on different aspects of health (Carstairs, 1981*a*; Townsend et al, 1988). In particular, studies using enumeration districts, rather than wards or other larger areas (Carstairs, 1981*b*), are considered more reliable since they tend to have more socially homogenous populations and their small size allows greater geographical resolution (Reading and Openshaw, 1993). There are, however, some acknowledged sources of inaccuracy in this approach. These include social heterogeneity within enumeration districts (Carr-Hill and Sheldon, 1991), in which the average Townsend score may be unrepresentative of some individuals within the district, and the possibility of social change, either before or after a census (Carr-Hill and Sheldon, 1991). There has also been some controversy over whether the census-derived deprivation scores actually measure an area's experience of material deprivation (Morris and Carstairs, 1991). We consider that despite their limitations for categorizing individual patients or areas, the Townsend scores have proven value for indicating average experience. Nonetheless, we could obtain information on material deprivation for patients only at the time of diagnosis. It may, of course, be that the frequency of EBV positivity in HD is affected by socioeconomic factors that are not necessarily present at the time of diagnosis, but which were extant some time prior to this, possibly during childhood.

### CONCLUSION

Our study has indicated that socioeconomic factors that seemingly account for differences in the relative proportions of EBV-positive and EBV-negative HD between countries, also appear to play a role in determining the frequency of EBV positivity within the UK. It is not known whether socioeconomic conditions affect the rate or timing of primary EBV infection, which may ultimately influence the likelihood of EBV positivity in HD. Alternatively, material deprivation may contribute to a decline in EBV-specific immunity, which may in turn also favour the development of EBV-positive HD.

### ACKNOWLEDGEMENTS

We are grateful to Lucinda Billingham, CRC Institute for Cancer Studies, University of Birmingham, for expert advice on the use of Townsend scores and to Adrian Harrison, Steven Hay and Steven Jones for their help in the collection of patient data.

### REFERENCES

Alexander FE, Daniel CP, Armstrong AA, Clark DA, Onions DE, Cartwright RA and Jarrett RF (1995) Case clustering, Epstein–Barr virus Reed–Sternberg cell status and herpes virus serology in Hodgkin's disease: results of a case control study. *Eur J Cancer* **31A**: 1479–1486

Ambinder RF, Browning PJ, Lorenzana I, Leventhal BG, Cosenza H, Mann RB, MacMahon EM, Medina R, Cardona V, Grufferman S, Olshan A, Levin A, Petersen EA, Blattner W and Levine PH (1993) Epstein–Barr virus and childhood Hodgkin's disease in Honduras and the United States. *Blood* **81**: 462–467

Anagnostopoulos I, Herbst H, Niedobitek G and Stein H (1989) Demonstration of monoclonal EBV genomes in Hodgkin's disease and Ki-1-positive anaplastic large cell lymphoma by combined Southern blot and in situ hybridization. *Blood* **74**: 810–816

Armstrong AA, Alexander FE, Cartwright R, Angus B, Krajewski AS, Wright DH, Brown I, Lee F, Kane E and Jarrett RF (1998*a*) Epstein–Barr virus and Hodgkin's disease: further evidence for the three disease hypothesis. *Leukemia* **12**: 1272–1276

Armstrong AA, Shield L, Gallagher A and Jarrett RF (1998*b*) Lack of involvement of known oncogenic DNA viruses in Epstein–Barr virus-negative Hodgkin's disease. *Br J Cancer* **77**: 1045–1047

Barletta JM, Kingma DW, Charache P, Mann RB and Ambinder RF (1993) Rapid in situ hybridization for the diagnosis of latent Epstein–Barr virus infection. *Mol Cell Probe* **7**: 105–109

Bernard SM, Cartwright RA, Darwin CM, Richards IDG, Roberts B, O'Brien C and Bird CC (1987) Hodgkin's disease: case-control epidemiological study in Yorkshire. *Br J Cancer* **55**: 85–90

Bithell JF, Dutton SJ, Neary NM and Vincent TJ (1995) Controlling for socioeconomic confounding using regression methods. *J Epidemiol Commun H* **49**: S15–S19

Carr-Hill R and Sheldon T (1991) Designing a deprivation payment for general practitioners: the UPA (8) wonderland. *Br Med J* **302**: 393–396

Carstairs V (1981*a*) Small area analysis and health service research. *Community Medicine* **3**: 131–139

Carstairs V (1981*b*) Multiple deprivation and health state. *Community Medicine* **3**: 4–13

Chang KL, Albujar PF, Chen Y-Y, Johnson RM and Weiss LM (1993) High prevalence of Epstein–Barr virus in the Reed–Sternberg cells of Hodgkin's disease occurring in Peru. *Blood* **81**: 496–501

Correa P and O'Conor GT (1971) Epidemiologic patterns of Hodgkin's disease. *Int J Cancer* **8**: 192–201

Frizzera G (1992) The distinction of Hodgkin's disease from anaplastic large cell lymphoma. *Semin Diagn Pathol* **9**: 291–296

Glaser SL, Lin RJ, Stewart SL, Ambinder RF, Jarrett RF, Brousset P, Pallesen G, Gulley ML, Khan G, O'Grady J, Hummel M, Preciado MV, Knecht H, Chan JK and Claviez A (1997) Epstein-Barr virus-associated Hodgkin's disease: epidemiologic characteristics in international data. *Int J Cancer* **70**: 375–382

Gulley ML, Eagan PA, Quintanilla-Martinez L, Picado AL, Smir BN, Childs C, Dunn CD, Craig FE, Williams JW Jr and Banks PM (1994) Epstein-Barr virus DNA is abundant and monoclonal in the Reed-Sternberg cells of Hodgkin's disease: association with mixed cellularity subtype and Hispanic American ethnicity. *Blood* **83**: 1595–1602

Gutensohn N and Cole P (1980) Epidemiology of Hodgkin's disease. *Semin Oncol* **7**: 92–102

© Cancer Research Campaign 1999 

Gutensohn N and Cole P (1981) Childhood social environment and Hodgkin's disease. *New Engl J Med* **304**: 135–140
Hansmann ML and Kuppers R (1996) Pathology and 'molecular histology' of Hodgkin's disease and the border to non-Hodgkin's lymphomas. *Baillière Clin Haematal* **9**: 459–477
Harris NL, Jaffe ES, Stein H, Banks PM, Chan JKC, Cleary ML, Delsol G, De Wolf-Peeters C, Falini B, Gatter KC, Grogan TM, Isaacson PG, Knowles DM, Mason DY, Muller-Hermelink H, Pileri SA, Piris NA, Ralfkiaer E and Warnke RA (1994) A revised European–American classification of lymphoid neoplasms: a proposal from the International Lymphoma Study Group. *Blood* **84**: 1361–1392
Herbst H, Dallenbach F, Hummel M, Niedobitek G, Finn T, Young LS, Rowe M, Muller-Lantzsch N and Stein H (1991) Epstein–Barr virus DNA and latent gene products in Ki-1 (CD30)-positive anaplastic large cell lymphomas. *Blood* **78**: 2666–2673
Herbst H, Steinbrecher E and Niedobitek G (1992) Distribution and phenotype of Epstein-Barr virus-harboring cells in Hodgkin's disease. *Blood* **80**: 484–491
Hummel M, Anagnostopoulos I, Dallenbach F, Korbjuhn P, Dimmler C and Stein H (1992) EBV infection patterns in HD and normal lymphoid tissue: expression and cellular localization of gene products. *Br J Haematol* **82**: 689–694
Jarrett RF, Gallagher A, Jones DB, Alexander FE, Krajewski AS, Kelsey A, Adams J, Angus B, Gledhill S, Wright DH, Cartwright RA and Onions DE (1991) Detection of Epstein–Barr virus genomes in Hodgkin's disease: relation to age. *J Clin Pathol* **44**: 844–848
Jarrett AF, Armstrong AA and Alexander E (1996) Epidemiology of EBV and Hodgkin's disease. *Ann Oncol* **7**: S5–S10
Leoncini L, Spina D, Nyong'o A, Abinya O, Minacci C, Disanto A, De Luca F, De Vivo A, Sabattini E, Poggi S, Pileri S and Tosi P (1996) Neoplastic cells of Hodgkin's disease show differences in EBV expression between Kenya and Italy. *Int J Cancer* **65**: 781–784
Levine PH, Pallesen G, Ebbesen P, Harris N, Evans AS and Mueller N (1994) Evaluation of Epstein-Barr virus antibody patterns and detection of viral markers in the biopsies of patients with Hodgkin's disease. *Int J Cancer* **59**: 48–50
Linden MD, Fishleder AJ, Katzin WE and Tubbs RR (1988) Absence of B-cell or T-cell clonal expansion in nodular lymphocyte predominant Hodgkin's disease. *Hum Pathol* **19**: 591–594
MacMahon B (1966) Epidemiology of Hodgkin's disease. *Cancer Res* **26**: 1189–1200
Morris R and Carstairs V (1991) Which deprivation? A comparison of selected deprivation indexes. *J Public Health Med* **13**: 318–326
Mueller N, Evans A, Harris NL, Comstock GW, Jellum E, Magnus K, Orentreich N, Polk BF and Vogelman J (1989) Hodgkin's disease and Epstein–Barr virus. Altered antibody pattern before diagnosis. *New Engl J Med* **32**: 689–695
Muir KR, Parkes SE, Mann JR, Stevens MCG and Cameron AH (1992) Childhood cancer in the West Midlands: incidence and survival, 1980–1984, in a multi-ethnic population. *Clin Oncol* **4**: 177–182
Murray PG, Young LS, Rowe M and Crocker J (1992) Immunohistochemical demonstration of the Epstein–Barr virus-encoded latent membrane protein in paraffin sections of Hodgkin's disease. *J Pathol* **166**: 1–5
Murray PG, Swinnen LJ, Constandinou CM, Pyle JM, Carr TJ, Hardwick JM and Ambinder RF (1996*a*) *bcl*-2 but not the EBV-encoded *bcl*-2 homologue, BHRF1, is commonly expressed in post-transplantation lymphoproliferative disorders. *Blood* **87**: 706–711
Murray PG, Billingham L, Devey EC, Kerr D, Crocker J and Young LS (1996b) The association between Epstein–Barr virus and Hodgkin's disease: correlation with survival and response to therapy. *J Pathol* **178**: 38A
Pallesen G, Hamilton-Dutoit SJ, Rowe M and Young LS (1991) Expression of Epstein–Barr virus latent gene products in tumour cells of Hodgkin's disease. *Lancet* **337**: 320–322
Reading R and Openshaw S (1993) Do inaccuracies in small area deprivation analyses matter? *J Epidemiol Commun H* **47**: 238–241
Shimkin MB (1955) Hodgkin's disease. Mortality in the United States, 1921–1951: race, sex, age distribution; comparison with leukemia. *Blood* **10**: 1214–1227
Stein H, Herbst H, Anagnostopoulos I, Niedobitek G, Dallenbach F and Kratzsch HC (1991) The nature of Hodgkin and Reed–Sternberg cells, their association with EBV, and their relationship to anaplastic large-cell lymphoma. *Ann Oncol* **2**: 33–38
Stiller CA, McKinney PA, Bunch KJ, Bailey CC and Lewis IJ (1991) Childhood cancer and ethnic group in Britain: a United Kingdom Children's Cancer Study Group (UK CCSG) Study. *Br J Cancer* **64**: 543–548
Stoler MH, Nichols GE, Symbula M and Weiss LM (1995) Lymphocyte predominance Hodgkin's disease – evidence for a kappa light chain-restricted monotypic B-cell neoplasm. *Am J Pathol* **146**: 812–818
Townsend P, Phillimore P and Beattie A (1988) *Health and Deprivation*: *Inequality and the North*. Croom Helm: London
Varghese C, Barrett JH, Johnston C, Shires M, Rider L and Forman D (1996) High risk of lymphomas in children of Asian origin: ethnicity or confounding by socioeconomic status? *Br J Cancer* **74**: 1503–1505
Weinreb M, Day PJR, Niggli F, Green EK, Nyongo'o AO, Othieno-Abinya NA, Riyat MS, Rafaat F and Mann JR (1996*a*) The consistent association between Epstein-Barr virus and Hodgkin's disease in children in Kenya. *Blood* **87**: 3828–3836
Weinreb M, Day PJR, Niggli F, Powell JE, Raafat F, Hesseling PB, Schneider JW and Hartely PS (1996*b*) The role of Epstein–Barr virus in Hodgkin's disease from different geographical areas. *Arch Dis Child* **74**: 27–31
Weiss LM, Strickler JG, Warnke RA, Purtilo DT and Sklar J (1987) Epstein–Barr viral DNA in tissues of Hodgkin's disease. *Am J Pathol* **129**: 86–91
Weiss LM, Chen YY, Liu XF and Shibata D (1991) Epstein–Barr virus and Hodgkin's disease: a correlative in situ hybridization and polymerase chain reaction study. *Am J Pathol* **139**: 1259–1265
Wu TC, Mann RB, Charache P, Hayward SD, Staal S, Lambe BC and Ambinder RF (1990) Detection of EBV gene expression in Reed–Sternberg cells of Hodgkin's disease. *Int J Cancer* **46**: 801–804
Zhou ZG, Hamilton-Dutoit S, Yan QH and Pallesen G (1993) The association between Epstein–Barr virus and Chinese Hodgkin's disease. *Int J Cancer* **55**: 359–363

*© Cancer Research Campaign 1999*
